# Staphylococcus lugdunensis Infectious Endocarditis Complicated by Embolic Stroke After Colonoscopy in a 58-Year-Old Female

**DOI:** 10.7759/cureus.24572

**Published:** 2022-04-28

**Authors:** Rafsan Ahmed, Mahmoud Alsaiqali, Asher Gorantla, Shruthi Sivakumar, Michelle Feinberg, Suzette Graham-Hill, Louis Salciccioli

**Affiliations:** 1 Department of Internal Medicine, State University of New York Downstate Medical Center, New York City, USA; 2 Department of Neurology, State University of New York Downstate Medical Center, New York City, USA; 3 Department of Neurosurgery, Kings County Hospital Center, New York City, USA; 4 Department of Cardiology, Kings County Hospital Center, New York City, USA; 5 Department of Cardiology, State University of New York Downstate Medical Center, New York City, USA

**Keywords:** prophylactic antibiotics prior to colonoscopy, embolic stroke, staphylococcus lugdunensis, infective endocarditis, post-colonoscopy bacteremia

## Abstract

There are a significant number of colonoscopies and esophagogastroduodenoscopies (EGDs) done in the United States every year and post-endoscopic infections are frequently seen. Data demonstrating causality between endoscopic procedures and infectious endocarditis (IE) or that antibiotic prophylaxis prior to endoscopic procedures protects against IE is still lacking. Here we have presented the case of a patient who underwent diagnostic colonoscopy as part of a malignancy workup and was later found to be septic with *Staphylococcus lugdunensis* bacteremia and had IE. We hypothesized that the infection was most likely contracted during colonoscopy as a result of bacterial translocation from the perineal region to the bloodstream. This case report highlights the need for further studies investigating the efficacy of prophylactic antibiotics in reducing the risk of IE after colonoscopies.

## Introduction

There are over 15 million colonoscopies and seven million esophagogastroduodenoscopies (EGDs) done in the United States every year [1,2]. Wang et al. conducted a study to estimate the rates of post-endoscopic infection in the ambulatory surgery setting and found that the incidence was more than one per 1000 screening colonoscopies and more than three per 1000 EGDs [3]. Another study, aiming to investigate rates of pneumonia and bacteremia after colonoscopy, found the seven-day pneumonia rate and seven-day bacteremia rate to be 2.7-4.4 per 10,000 procedures and 2.9-3 per 10,000 procedures, respectively [4]. In a review by Nelson et al., it was shown that rates of bacteremia associated with colonoscopy have been as high as 25% with a mean of 4.4% [5]. Post-endoscopic bacteremia has been advocated as a surrogate marker for infectious endocarditis (IE) risk and only 25 cases of IE have been reported with a temporal association to an endoscopic procedure [6-8]. Data demonstrating causality between endoscopic procedures and IE or that antibiotic prophylaxis prior to endoscopic procedures protects against IE is still lacking. We present a case of a patient who developed infective endocarditis from *Staphylococcus lugdunensis* (*S. lugdunensis*) after a diagnostic colonoscopy. 

## Case presentation

A 58-year-old African American female was found unresponsive at home on June 29, 2021, and was brought in by emergency medical services to our hospital after being intubated in the field due to a Glasgow coma scale of 3. She had a past medical history of human immunodeficiency virus (HIV), on anti-viral medication with CD4 count of 625 and undetectable viral load, type 2 diabetes mellitus, hypertension, and schizophrenia. Of note, she was admitted to a different medical center on June 15, 2021, for left femoral deep venous thrombosis and bilateral pulmonary embolism for which she was started on anticoagulation therapy; no blood cultures were collected at the time. Computed tomography angiography (CTA) of the chest also incidentally revealed hepatic lesions suspicious for metastatic disease. During that admission the patient was also seen by gastroenterology service and she underwent a colonoscopy on 22 June, 2021; a polyp was removed via cold snare polypectomy in the ascending colon with minimal blood loss. Pathology was remarkable for tubular adenoma. It is also important to mention that she denied ever using intravenous drugs and that she did not have any invasive vascular procedures done, e.g. central line placement, during that admission. She was discharged home on June 24, 2021, with outpatient follow-up with interventional radiology for liver lesion biopsy. 

**Figure 1:**
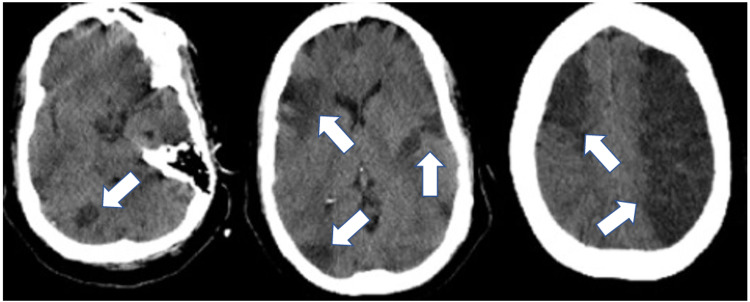
CT scan of the head at different axial sections shows multiple vascular territory infarcts (white arrows)

**Figure 2:**
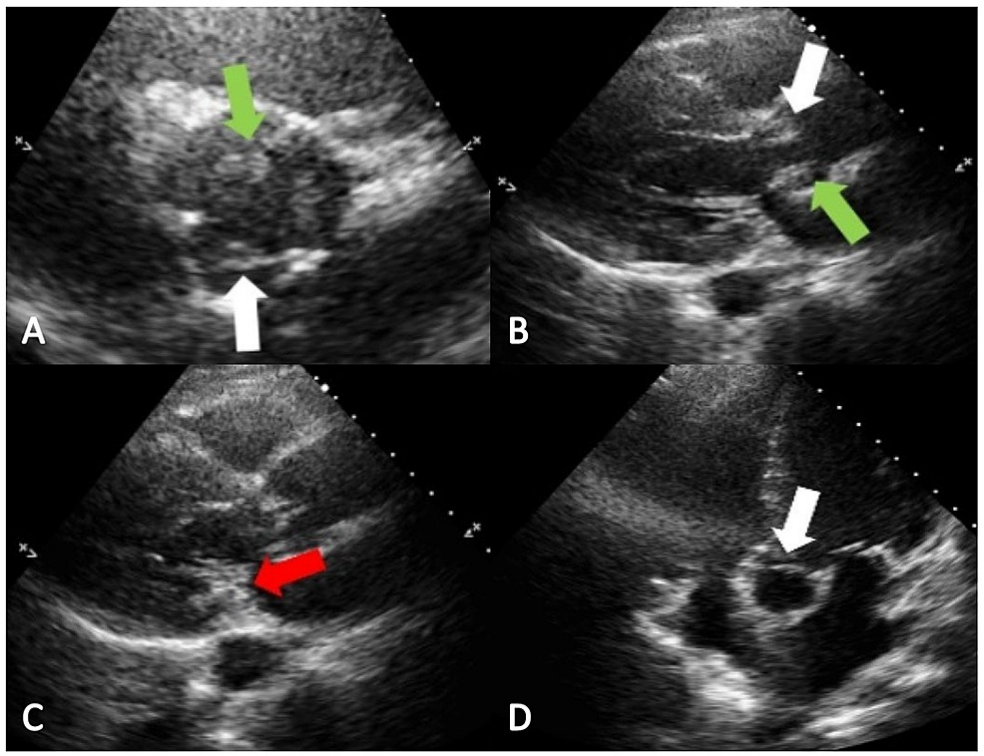
Transthoracic echocardiogram: (A) Parasternal-short axis view shows mobile hypodensity in the noncoronary cusp (green arrow) and in the right coronary cusp (white arrow); (B) Parasternal-long axis view shows mobile hypodensity in the noncoronary cusp (green arrow) and in the right coronary cusp (white arrow); (C) Parasternal-long axis view that shows mobile hypodensity in the posterior mitral leaflet (red arrow); (D) Apical five chamber view shows mobile hypodensity in the right coronary cusp (white arrow)

On presentation to our hospital, her vital signs were significant for a temperature of 103.1^o^F, heart rate of 137 beats per minute, and blood pressure was 150/101 mmHg. The initial lab values are represented in Table 1. She was started on broad spectrum antibiotic coverage with vancomycin and piperacillin-tazobactam. CT head showed an intraparenchymal hemorrhage in the temporoparietal lobe with surrounding edema, as well as multiple vascular territory infarcts that are subacute to chronic consistent with an embolic process (Figure 1). The patient was admitted to the neurosurgery intensive care unit (NSICU) for further management. Transthoracic echocardiogram (TTE) showed new findings of severe aortic regurgitation, mobile echodensity on the posterior mitral leaflet, and mobile echodensity on the inferior surface of the aortic valve that was highly suggestive of vegetation (Figure 2). This finding was not evident on a TTE done on June 15, 2021, to evaluate right heart strain during her previous admission. Blood cultures grew *S. lugdunensis* raising concern for acute IE. *S. lugdunensis* was resistant to azithromycin, clindamycin, erythromycin, and penicillin only.

**Table 1:** Initial lab values on admission

Laboratory Test	Value on admission	Reference range
White blood cell (WBC) count	24,000 (× 10^3/microL)	3.8 to 10.4 (× 10^3/microL)
Creatinine	2.3 mg/dL	0.6 - 1.3 mg/dL
Troponin	2.2ng/mL	0 - 0.04 ng/mL

The patient’s hospital course was complicated by hypotension, persistent leukocytosis in the setting of fevers, and further worsening of kidney function. The patient was started on vasopressors, and the antibiotic regimen was modified; vancomycin and piperacillin-tazobactam were switched to daptomycin and meropenem due to worsening renal function. Later blood cultures showed no further growth of bacteria and the patient showed initial improvement; however, she worsened soon after vasopressors were discontinued. Cardiology service was consulted for IE but the patient was not a candidate for cardiothoracic surgery given her medical and functional status. 

Repeat CT head showed mass effect and growing areas of infarction. The medical team held a family meeting during which the patient’s poor prognosis was explained to the family. After a prolonged discussion for several days and further worsening of clinical status, the family decided to pursue comfort care. The patient was terminally extubated after which she expired. 

## Discussion

In our case, the patient presented with sepsis and was found to have an embolic stroke with echocardiographic evidence of endocarditis and blood cultures growing *S. lugdunensis*. *S. lugdunensis*, which was first described in 1988, is a gram-positive coagulase-negative staphylococcus (CoNS) and a member of normal skin commensals, preferentially colonizing the perineal region of the body [9]. The true prevalence of *S. lugdunensis* is unknown as few laboratories speciate CoNS. In a study by Gatermann et al., among 494 clinical isolates of CoNS, *S. lugdunensis* accounted for 3% of isolates [10]. It is a rare but destructive cause of endocarditis and has been implicated in both native and prosthetic valve endocarditis. Identifiable sources of infection include perineal surgery, vascular access, pacemaker insertion sites, and skin [11]. Embolic sequelae of *S. lugdunensis* causing cerebrovascular events are rare and only a few cases have been reported [12]. The majority of IE caused by *S. lugdunensis *involve native valves, mitral and aortic valves are often reported, and in some cases, multivalve endocarditis is also reported (as seen in our patient) [13]. Treatment of native valve *S. lugdunensis* IE consists of six weeks of parenteral beta-lactam therapy or vancomycin depending on sensitivity studies [14]. In a review of 10 patients with *S. lugdunensis* IE, 60% of patients with left-sided IE required surgery after experiencing serious complications such as heart failure, periannular abscess, and shock [14]. Predictors of surgery in *S. lugdunensis* IE include age less than 50 years, absence of significant comorbidity, aortic valve involvement, and periannular abscess [14]. Liu et al. stated in their review that *S. lugdunensis* IE was associated with a 38% mortality rate and surgery was needed in 68% of cases [11]. Takahashi et al. reported an 18% decrease in *S. lugdunensis* IE-related mortality after 1993 due to early recognition and timely surgical intervention [15].

In our patient, we hypothesized that the infection was most likely contracted during colonoscopy as a result of bacterial translocation from the perineal region to the bloodstream during the polypectomy or due to a mucosal tear during the procedure. A case report of embolic stroke caused by *S. lugdunensis* IE after vasectomy also shows that procedures involving instrumentation of the perineal region may lead to this systemic infection [16]. Additionally, the patient’s compromised immune system as a result of HIV infection increased her likelihood of bloodstream infection [17]. The American Heart Association (AHA) recognizes patients with prosthetic cardiac valves, a history of previous IE, cardiac transplant recipients who develop valvulopathy, and patients with congenital heart disease to have the highest risk of an adverse outcome from IE, and suggests the use of an antibiotic effective against enterococci prior to undergoing an endoscopic procedure in individuals with established gastrointestinal (GI) tract infections [18]. However, the recent AHA guidelines do not recommend the administration of prophylactic antibiotics solely to prevent IE for patients undergoing GI endoscopy [18]. 

## Conclusions

We have presented a case of a patient who underwent diagnostic colonoscopy as part of a malignancy workup and was later found to be septic with *S. lugdunensis* bacteremia and had IE. Her hospital course was further complicated by embolic stroke and further deterioration, which eventually led to her demise. *S. lugdunensis* IE is a rare entity in itself, however, there are no reports of this infection being acquired after a colonoscopy procedure. The present literature on causality between endoscopic procedures and IE or whether prophylactic antibiotics prior to endoscopic procedure protects against IE is very limited. This case report highlights the need for further studies investigating the efficacy of prophylactic antibiotics in reducing risk of IE after colonoscopies. 
